# New York State Board of Medical Examiners

**Published:** 1895-09

**Authors:** 


### NEW YORK STATE BOARD OF MEDICAL EXAMINERS.

THE first meeting of the State Board of Veterinary Medical Examiners was held in the Regents' office at the Capitol, in Albany, July 25, 1895. The Board was organized by the election of Professor James Law, of Cornell University, President, and William H. Kelly, of Albany, Secretary.

After discussion the subjects were grouped as follows: 1, anatomy and surgery; 2, physiology and hygiene; 3, chemistry. therapeutics, and materia medica; 4, obstetrics; 5, pathology, diagnosis, and practice.

By the President's appointment the members of the Board were assigned chairs as follows: Dr. R. S. Huidekoper, anatomy and surgery; Dr. C. D. Morris, physiology and hygiene; Dr. N. P. Hinkley, chemistry, therapeutics, and materia medica; Dr. W. H. Kelly, obstetrics; Professor James Law, pathology, diagnosis, and practice.

It was resolved that a syllabus be drafted, each member of the Board to prepare that part of the same which pertains to his chair, and the first draft to be submitted to the Regent's office before August 4th.

It was voted that at the meeting of the Board in September, which, it is understood, the President shall call about the 5th, each member is to submit to the Board a set of fifteen questions on each of the following subjects: 1, comparative anatomy; 2, chemistry; 3, obstetrics; 4, therapeutics and materia medica; 5, physiology and hygiene; 6, veterinary surgery; 7, pathology, diagnosis, and practice.

It was voted that Dr. R. S. Huidekoper be the questions editor, and that proof of all questions for examinations be submitted to him before they are finally printed by the Regents.

It was voted that at the September meeting each member shall bring a list of the veterinary schools and colleges which he deems worthy, by reason of prescribed courses of study, to be registered by the Board.

It was voted that the whole Board shall act as a committee on examination questions.

It was voted that in the intervals between the meetings of the Board any decision or resolution signed by all the members of the Board shall be official.

The meeting adjourned subject to the call of the President.

